# Supplementary material for: Parkinson Disease Protein 7 (PARK7) Is Related to the Ability of Mammalian Sperm to Undergo In Vitro Capacitation
Source: Int J Mol Sci. 2021 Oct 6;22(19):10804. doi: 10.3390/ijms221910804 (PMC8509420; doi:10.3390/ijms221910804)
Supplement: Supplementary file 1 [file ijms-22-10804-s001.zip › Recuero et al 2021_SupplementaryMaterial.pdf]

Supplementary material (Recuero et al.)

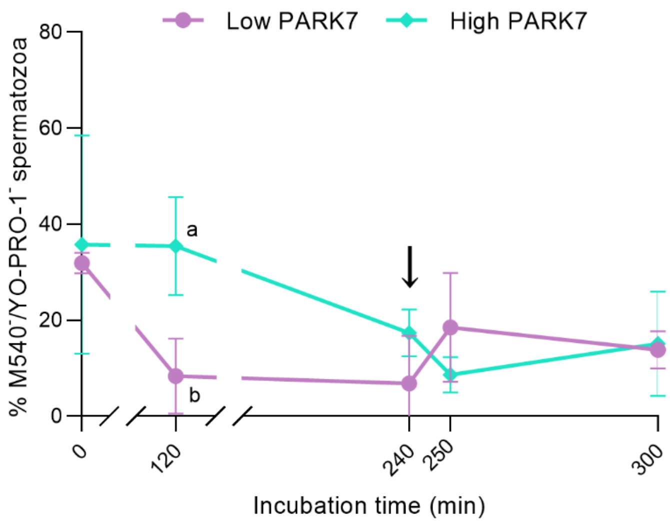

Figure S1. Percentage of viable spermatozoa with low lipid membrane disorder ($M540^-$/YO-PRO-1^-) during in vitro capacitation and progesterone-induced acrosome exocytosis (300 min). Black arrow indicates the time at which progesterone was added at a final concentration of 10 µg/mL (240 min). Data are shown as mean ± SD. Different letters indicate significant differences (p < 0.05) between samples containing high or low levels of PARK7 at 0 min of incubation.

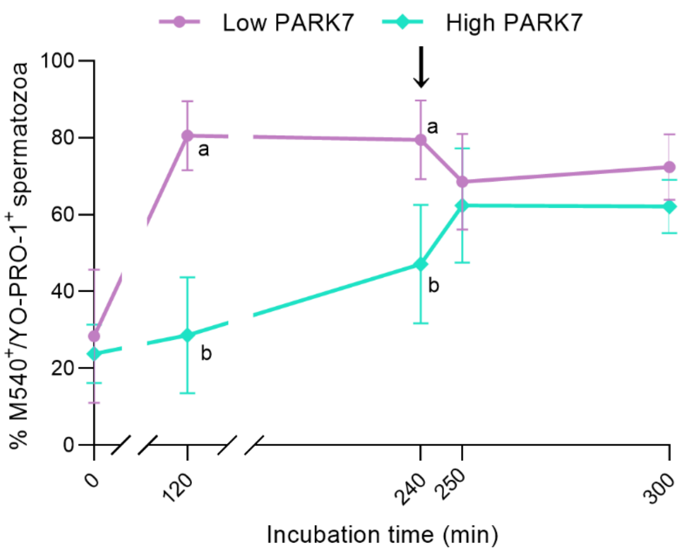

Figure S2. Percentage of non-viable spermatozoa with high lipid membrane disorder ($M540^+$/YO-PRO-1^+) during in vitro capacitation and progesterone-induced acrosome exocytosis (300 min). Black arrow indicates the time at which progesterone was added at a final concentration of 10 µg/mL (240 min). Data are shown as mean ± SD. Different letters indicate significant differences (p < 0.05) between samples containing high or low levels of PARK7 at 0 min of incubation.

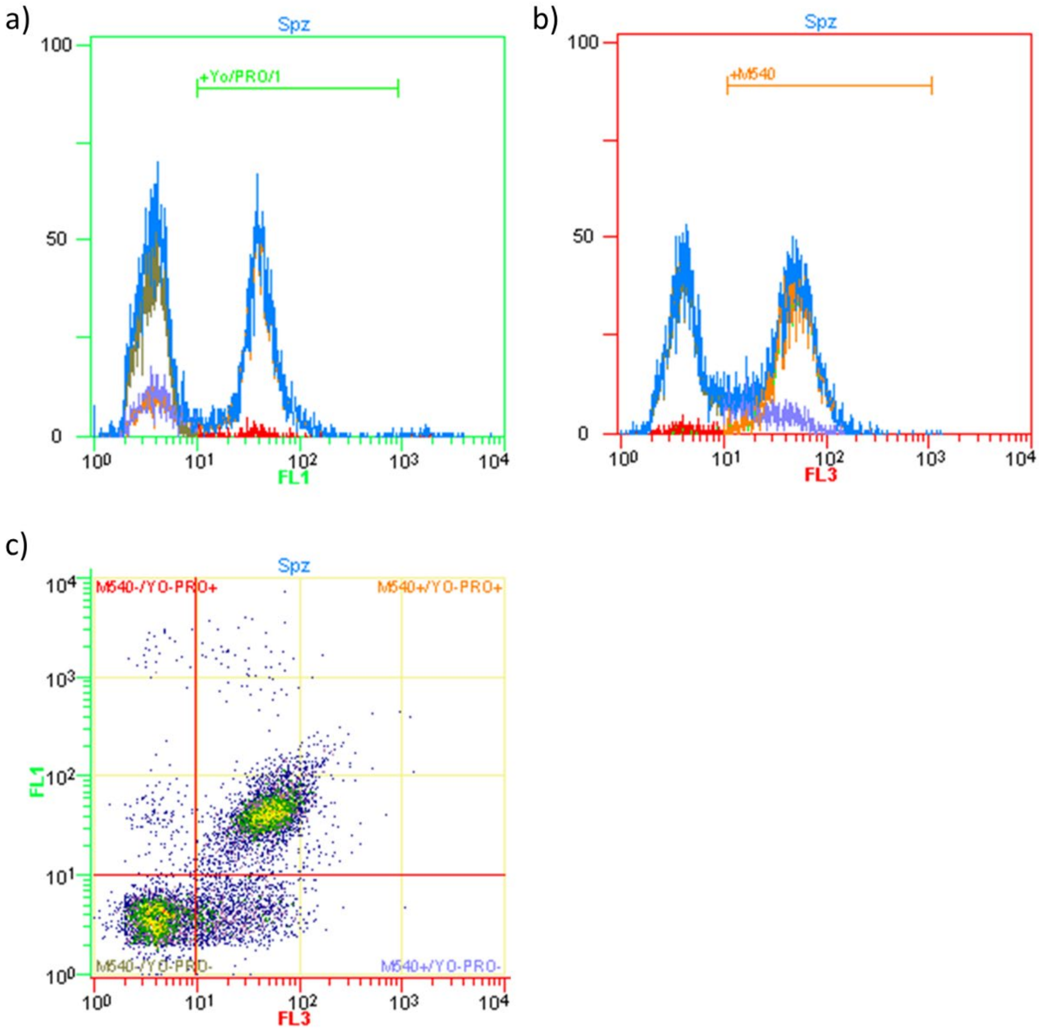

Figure S3. Representative flow cytometry YO-PRO (**a**) and M540 (**b**) histograms of sperm samples at the beginning of the in vitro capacitation experiment (0 min of incubation). **(c)** Dot plots of M540 and YO-PRO staining showing four distinct populations: M540$^-$/YO-PRO$^+$, M540$^+$/YO-PRO$^-$, M540$^-$/YO-PRO$^-$, and M540$^+$/YO-PRO$^+$.

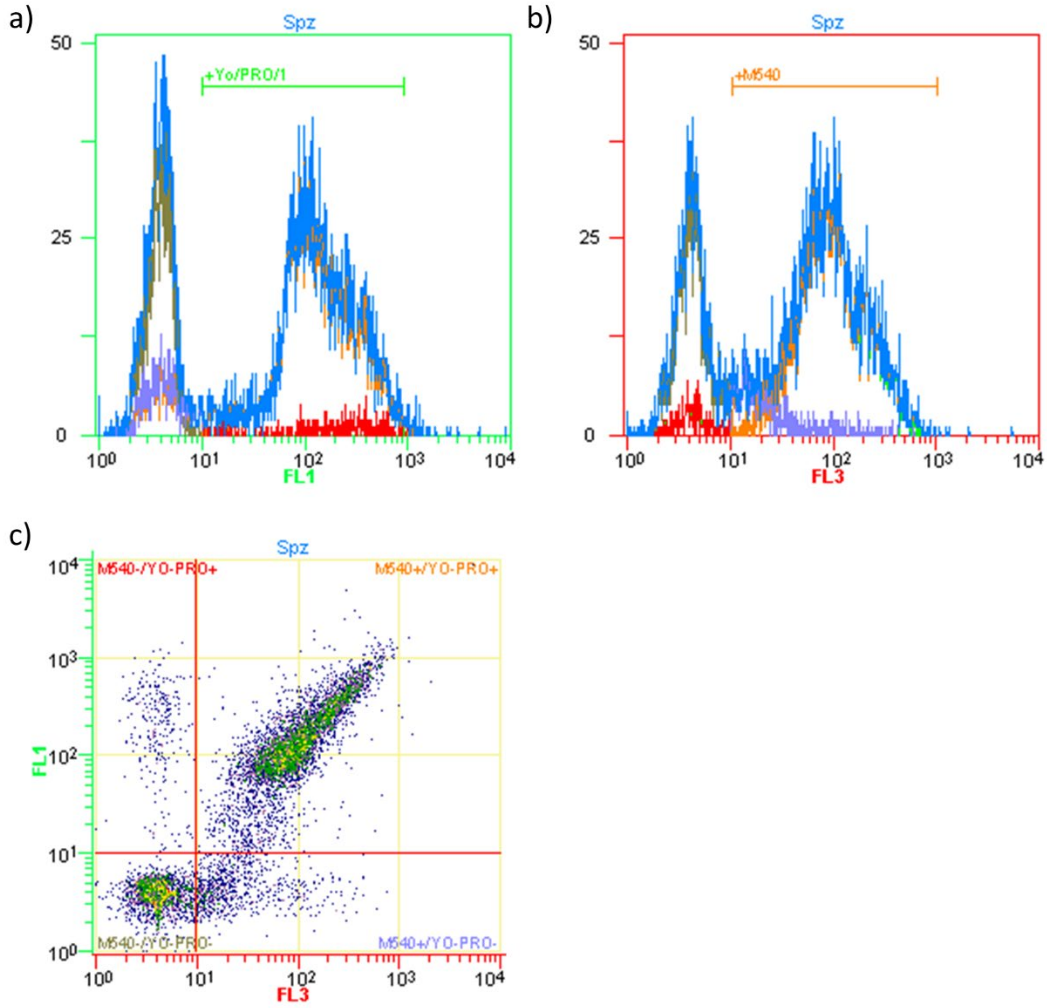

Figure S4. Representative flow cytometry YO-PRO (**a**) and M540 (**b**) histograms of sperm samples after 240 min of incubation in capacitation medium. **(c)** Dot plots of M540 and YO-PRO staining showing four distinct populations: M540$^-$/YO-PRO$^+$, M540$^+$/YO-PRO$^-$, M540$^-$/YO-PRO$^-$, and M540$^+$/YO-PRO$^+$.

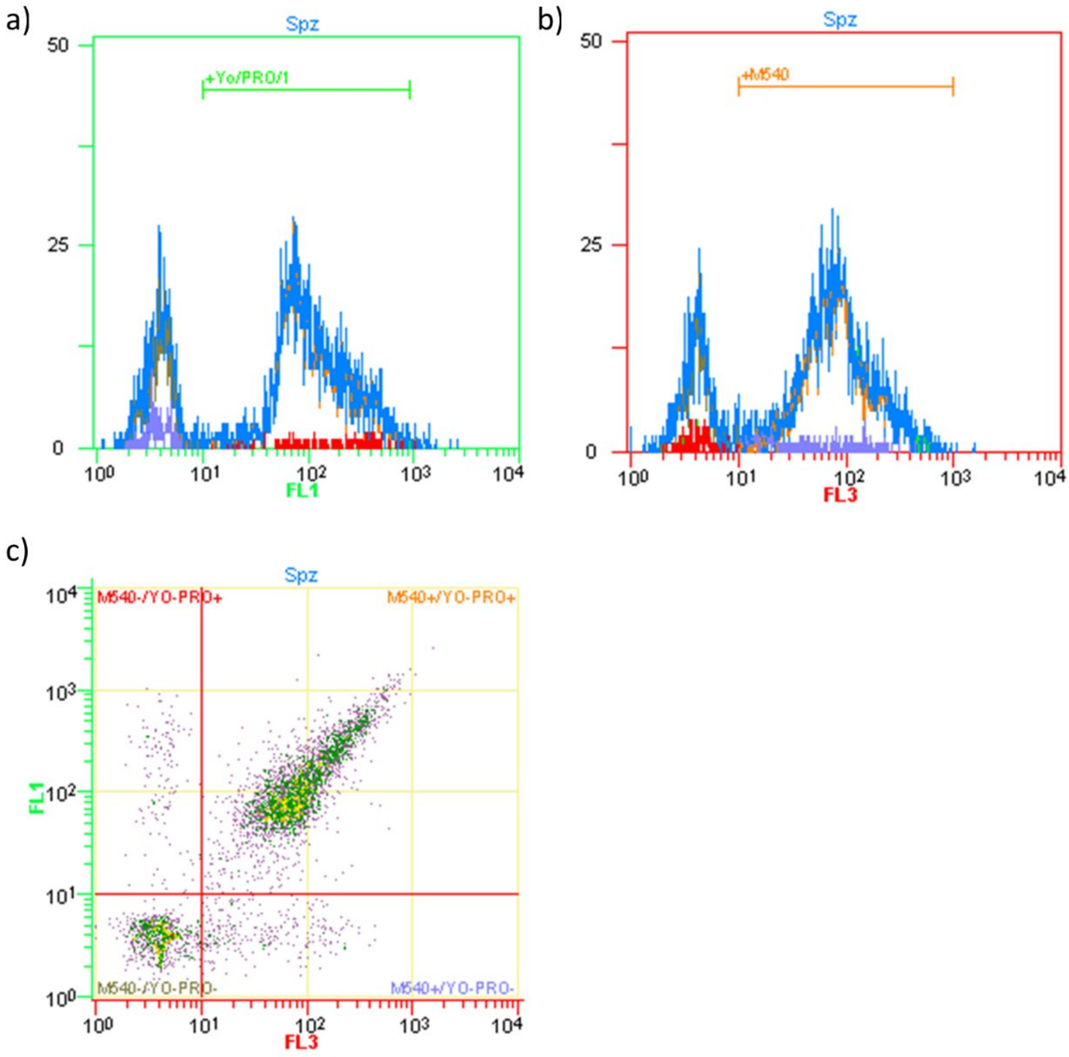

Figure S5: Representative flow cytometry YO-PRO (**a**) and M540 (**b**) histograms of sperm samples after 300 min of incubation in capacitation medium. **(c)** Dot plots of M540 and YO-PRO staining showing four distinct populations: M540^{-}/YO-PRO^{+}, M540^{+}/YO-PRO^{-}, M540^{-}/YO-PRO^{-}, and M540^{+}/YO-PRO^{+}.

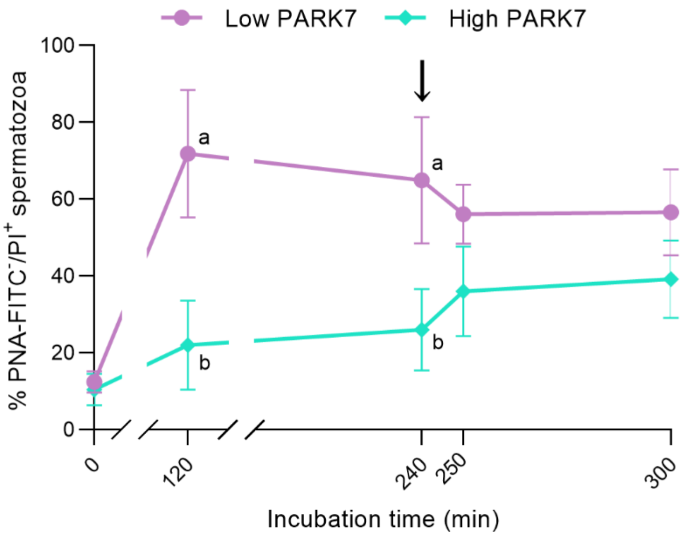

Figure S6: Percentage of non-viable spermatozoa with an acrosome that could not be fully intact (PNA-FITC+/PI+) during in vitro capacitation and progesterone-induced acrosome exocytosis (300 min). Black arrow indicates the time at which progesterone was added at a final concentration of 10 µg/mL (240 min). Data are shown as mean ± SD. Different letters indicate significant differences ($p < 0.05$) between sperm samples with high or low PARK7 levels at 0 min of incubation.

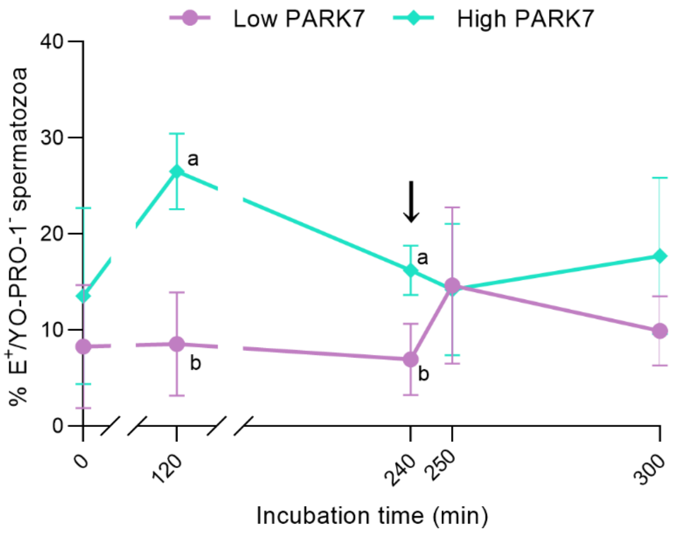

Figure S7: Percentage of viable sperm with high intracellular O_2^- levels (E^+/YO-PRO-1^-) during in vitro capacitation and progesterone-induced acrosome exocytosis (300 min). Black arrow indicates the time at which progesterone was added at a final concentration of 10 µg/mL (240 min). Data are shown as mean ± SD. Different letters indicate significant differences ($p < 0.05$) between sperm samples with high or low PARK7 levels at 0 min of incubation.
